# Sight or smell: which senses do scavenging raptors use to find food?

**DOI:** 10.1007/s10071-018-1220-0

**Published:** 2018-10-26

**Authors:** Simon Potier, Olivier Duriez, Aurélie Célérier, Jean-Louis Liegeois, Francesco Bonadonna

**Affiliations:** 1 CEFE UMR 5175, CNRS-Université de Montpellier-Université Paul-Valéry Montpellier-EPHE-1919 Route de Mende, 34293 Montpellier Cedex 5, France; 2 Department of Biology, Lund University, Sölvegatan 35, 22362 Lund, Sweden; 3 Académie de Fauconnerie du Grand Parc du Puy du Fou, CS 70 025, 85590 Les Epesses, France

**Keywords:** Foraging, Olfaction, Raptors, Southern caracara, Turkey vulture, Vision

## Abstract

Raptors are usually considered to be mainly visually dependent, and the use of other sensory modalities has rarely been studied in these birds. Here, we investigated experimentally which senses (vision and/or olfaction) Turkey vultures (*Cathartes aura*) and Southern caracaras (*Caracara plancus*) use to find hidden food. First, two identical stainless-steel perforated balls, one containing a putrefied piece of meat and the other an odorless control, were presented to birds in binary choice experiments. Both species interacted more with the smelling ball than with the control, suggesting that they were attracted by the odor of the hidden meat. In a second experiment, individuals were accustomed to eat in one specifically colored ball (blue or green). In the test phase, the meat was hidden in the opposite color with respect to the one each bird had become accustomed to. Vultures still interacted more with the smelly ball disregarding the color, while caracaras interacted equally with the two balls. The prevalence of olfaction in Turkey vultures may partly explain why they are the first raptors to find carcasses in tropical forests. In contrast, caracaras forage on the ground opportunistically, a strategy where both olfaction and sight may be involved. Our experiments suggest that both species are able to use olfactory cues for foraging. However, olfaction could be the predominant sense in Turkey vultures while olfaction and sight could play an equivalent role in Southern caracaras.

**Electronic supplementary material:**

The online version of this article (10.1007/s10071-018-1220-0) contains supplementary material, which is available to authorized users.

## Introduction

*Vox populi* often generalizes the sensory abilities of different animals. However, animals experience a variety of different ecological conditions throughout their lives, which may favor the use of different sensory abilities (Ruzicka and Conover 2012; Vander Wall et al. 2003), even in those organisms that “are known” to rely predominantly on one sense.

Historically, birds were considered to have a very poor or even non-existent sense of smell (Audubon 1826; Parsons 2013). Despite the work of a few pioneers in the 1970s who discovered that some bird species rely on olfaction for navigation (Papi et al. 1972) and sometimes for foraging (Wenzel 1971), birds were, and still are, predominantly considered to be visual foragers, even though species that live in environments where visual cues are limited (e.g., dense vegetation) or almost absent (e.g., oceans) could have evolved alternative sensory abilities such as olfaction (Nevitt et al. 1995). While it is now well accepted that many birds do indeed have a functional sense of smell (Caro et al. 2015), the relative importance of olfaction compared to other senses, such as vision, remains poorly studied, especially for a specific function such as foraging. Studies on the roles of vision and olfaction in feeding behavior in a variety of animals, including insects (Balkenius et al. 2006; Raguso and Willis 2002; Stöckl et al. 2016), snakes (Shivik and Clark 1997; Teather 1991), fish (Batty and Hoyt 1995; Webster et al. 2007) and mammals (Langley 1983; Raghuram et al. 2009; Wells and Lehner 1978) have shown the dual importance of these modalities, either independently or in combination. In birds, studies on nocturnal and oceanic birds suggest that, at least in some species, olfaction could play an important role in behavior. For instance, the North Island Brown kiwi, *Apteryx mantelli*, may rely on olfaction to localize profitable patches of food and then tactile information from a bill tip organ to locate individual items (Cunningham et al. 2009). Other examples may be found in procellariiform seabirds, which are generally considered excellent among birds in their use of olfactory cues. For example, Wandering albatrosses, *Diomedea exulans*, may use olfaction to detect prey at long distances and then rely on vision at short distances (Nevitt et al. 2008), but both visual and olfactory stimuli presented together elicit a higher probability of successfully finding food than separately in an experimental setup (Mardon et al. 2010). Furthermore, Cory’s and Scopoli’s shearwaters (*Calonectris borealis* and *C. diomedea*) can orientate using olfactory cues when available, but are thought to shift to use vision when deprived of their sense of smell (Dell’Ariccia and Bonadonna 2013; Gagliardo et al. 2013; Pollonara et al. 2015).

In contrast to procellariiformes, accipitriform and falconiform diurnal birds of prey (hereafter called raptors) are considered to be highly dependent on vision for survival. Indeed, these birds have relative large eyes (compare to body mass), well-developed fovea(s), high densities of cone photoreceptors and retinal ganglion cells, and the highest visual acuity found in animals (Hirsch 1982; Inzunza et al. 1991; Jones et al. 2007; Mitkus et al. 2018; Potier et al. 2016a, b, 2017; Reymond 1985, 1987). However, field observations suggest that two species of New World vultures (*Cathartes sp*., family Cathartidae) could also use olfactory cues to find decomposing carrion (Gomez et al. 1994; Graves 1992; Houston 1986, 1988; Smith and Paselk 1986). Furthermore, the olfactory bulbs in some raptors species, including Turkey vultures (Grigg et al. 2017), are relatively large (Zelenitsky et al. 2011), and functional olfactory genes have been found in these birds (Yang et al. 2015; Zhan et al. 2013). Nevertheless, evidence from behavioral experiments is still needed, even though a recent study showed that raptors from different species may be able to learn olfactory cues (Slater and Hauber 2017). However, as these authors grouped nine individuals from five species, they were not able to conclude which species used their sense of smell. Another study suggested that the Oriental honey buzzard *Pernis orientalis* could smell the pollen from the pollen dough provided by beekeepers to bees (Yang et al. 2015), but the experimental setup used did not allow clear conclusions to be made about the preferential use of olfaction or vision. Indeed, while all pollen dough had similar colors, the authors themselves declared that they could not be certain that removing the pollen from the pollen dough (to remove the odor of the pollen) did not change the texture (and therefore the visual appearance) of the dough. In summary, there is a distinct lack of behavioral studies designed to assess the olfactory abilities in diurnal raptors. It is important to understand whether raptors can functionally use their sense of smell for a specific function, such as foraging, navigation or communication, as already shown in other bird species (Nevitt et al. 1995; Wallraff 2005; Caro et al. 2015).

Here, we performed experiments to study the role of olfaction in foraging behavior in two opportunistic carrion-eating raptor species: The Turkey vulture *Cathartes aura* (Cathartidae: Accipitriformes) and the Southern caracara *Caracara plancus* (Falconidae: Falconiformes). Both species live in the same geographical area (Central and South America). While the Turkey vulture is an obligate scavenger, the Southern caracara is a generalist predator that eats carrion opportunistically, but which also forages on invertebrates and small vertebrates (Del Hoyo and Elliot 1994). These two species are relatively similar in terms of their size and their diet, but because of differences in their ecology, we predicted that they may rely differently on their visual and olfactory systems to find food. In the wild, Turkey vultures and Southern caracara forage in both open-field and forested environments. In open-field environments, food can be seen at distance by both species. By contrast, in forested environments, Turkey vultures search for food while flying over the forest, whose canopy hides food sources (Houston 1986). Thus, in forested environments, we predicted that in this species, olfaction may be more important than vision when detecting carcasses. In contrast, Southern caracaras forage on the ground opportunistically, moving under the canopy (Sazima 2007) where vision may be more important. However, in urbanized areas, they inspect, seemingly by sight, garbage bins, but surprisingly only open those plastic bags containing food even if those plastic bags are opaque (F. B. personal observation). In this situation, caracaras may hypothetically identify the correct bags using olfactory cues: for example, if the garbage bags are not well sealed, or because most of the plastic bags used for garbage may be permeable to odors. However, we cannot exclude an identification by associative learning of a non-olfactory stimulus present in the plastic bags. Thus, we suggest that caracaras may have a functional sense of smell that they can used to find food, but that they are not as reliant on olfaction for foraging as Turkey vultures are.

In this paper, we first tested the ability of both species to detect hidden food items by smell alone (experiment 1). Second, we tested the relative importance of vision and olfaction in an experimental paradigm involving both visual and olfactory cues at the same time (experiment 2).

## Materials and methods

The study was carried out using captive raptors housed at two bird parks in France; “Le Grand Parc du Puy du Fou” (site 1; experiments 1 and 2), and “Le Zoo d’Amnéville” (site 2; experiment 2). The first experiment involved five Turkey vultures and five Southern caracaras at site 1 (Table S1). The second experiment involved four of the same individuals for each species at site 1, plus four additional individuals of each species at site 2 (Table S1). The experiments were performed at different times of the year (April and October 2014) due to the availability of the birds at the two sites. We contacted all the French parks housing these species and used all the birds available in those parks that allowed us to perform the experiments.

### Ethics

The study was conducted under a formal agreement between the animal rearing facilities at Le Grand Parc du Puy du Fou and Le Zoo d’Amnéville, and Centre National de la Recherche Scientifique (CNRS). In agreement with French law, all the birds were handled by their usual trainer, under the permit of the Grand Parc du Puy du Fou (national certificate to maintain birds “Certificat de capacité” issued to the director of falconry, Jean-louis Liegeois, on 7 April 1994) and the Zoo d’Amnéville [national certificate to maintain birds “Certificat de capacité” issued to the director, Michel Louis, on 28 March 2007 (N° 2007-DEDD/BEN 03)].

### Experimental birds

Since hatching, or after 2 months in the case of two of the Turkey vultures from site 2, the experimental birds were hand-reared, and thus were familiar with humans. For site 1, each caracara was housed individually in an aviary each 6 × 4 × 4 m, while the Turkey vultures lived together in groups in a larger aviary measuring 20 × 8 × 6 m. For site 2, the caracaras and Turkey vultures were housed together in aviaries of 5 × 6 × 5 m and 5 × 7.5 × 3.5 m, respectively. All of the birds at both sites were fed with 3-day-old dead chicks once a day. During public shows, birds were brought to “flight condition”, a falconry term that refers to the theoretical weight of wild birds when they search for food. Thus, when in flight condition, the birds were not starving, but they were hungry enough to search for food. The falconers weighed every bird each day, to check their body condition and adjust their diet if necessary. Before the experiments, birds were being used in shows and were thus in flight condition. During the experiments, birds were fed only after each trial with the reward of the experiment. Birds did not participate in shows during the experiments. None of the experimental birds were in a reproductive state. After the experiments, the birds were returned to their daily training routines. At both sites, similar training routines were used. Falconers asked the birds to fly to their falconry glove using a visual cue (a fresh piece of chicken meat is placed in the glove).

### Olfactory stimuli (experiments 1 and 2)

For experiment 1, the olfactory stimuli were small pieces of meat from local butcher (beef, 20 ± 1 g), kept for 4 days at ambient temperature in a garbage bag (to make it more odorant), and then stored at 4 °C. The treatment affected to the meat was adjusted to control the putrefaction and thus the level of odorous gases produced by the decomposition. Putrefied meat appeared to be biologically significant for these birds as carrion is an important part of the diet for both species in the wild. We used beef instead of chicken because we wanted to highlight an olfactory sensibility regardless to memory processes such as habituation (chicken was used during the birds’ daily training routines by falconers and so using this food item could have had a confounding effect due to this previous association). Mean ambient temperatures were not statistically different between time of experiments in April and October 2014 (April 10.8 °C ± 1.1; October 11.7 °C ± 1.9 (mean ± se); Wilcoxon test, *N* = 12, *W* = 10, *p* = 0.69).

For experiment 2, birds came from two different sites (see Table S1) with different environmental conditions with regard to, for example, ambient temperature and hours of sunshine. Therefore, we changed the procedure used to prepare the meat (to adjust the decomposition level of meat in both experiments). Consequently, for experiment 2, the meat (again beef, 20 ± 1 g) was kept for 4 days at 15 °C precisely in a garbage bag, and then stored it at 4 °C.

To completely conceal the visual component of the olfactory stimulus (or the control, which consisted of a 20 g piece of new plastic that supposedly had no odor, or no biologically relevant odor for the foraging birds) without blocking the odor, the pieces of meat (or plastic) were inserted into small steel tea strainers 4 cm high and 2 cm wide (Birambeau, Paris, France), which were then placed inside larger stainless-steel rice cooking balls (perforated steel balls allowing odors diffusion, Städter, Allendorf, Germany; Fig. S1). A tea strainer was placed inside the rice cooking balls to prevent the birds from visually inspecting the contents of the balls by looking through the holes of the balls. We used two similar balls for each treatment. The balls had a diameter of 10.5 cm for experiment 1 and 14 cm for the experiment 2. The ball containing the olfactory stimulus is hereafter called the smelling ball, while the ball containing the control is hereafter called the control ball.

For 7 days prior to the experiments, the birds were fed with their standard food (dead chicks) in the open rice cooking balls to familiarize them to the devices. While on the first day individuals were not interested in the balls, they started to interact with the balls on the second days, once they learned (after the reward obtained the first day) that food item was placed inside. During both training sessions and experiments, the balls were not attached to the ground, and could be freely moved by the birds. For both experiments 1 and 2, we used different sets of balls for the training and the test session. The balls were thoroughly cleaned using alcohol and water after each trial.

### Experimental aviaries (experiments 1 and 2)

The experiments were performed in wood-made closed aviaries to avoid any attenuation of the olfactory stimuli by air currents or wind. The aviaries were closed on all four sides. For logistical reasons and availability of the facilities, two different closed aviaries were used, depending on the site. At both sites, the aviaries had similar dimensions (6 × 8 × 4 m for experiments 1 and 2 at site 1, and 5 × 7.5 × 3.5 m for experiment 2 at site 2) with sand on the floor. A starting perch (1 m high) was placed 4 m from the balls, which were placed 6 m apart. Each trial was filmed with a video-camera (GoPro Hero3+, San Mateo, California, USA) placed 3 m above the starting perch, which could record bird behavior over the entire aviary (Fig. S2). All birds were acclimated to the aviaries for 4 h the day before the test (this acclimation time was based on previous observations by experienced falconers). After each trial, the aviary floors were cleaned by hand and with a rake to avoid any biases, such as the footprints, dropping and feathers of the previous bird.

### Experiment 1: olfactory abilities

Each bird performed one 10-min trial per day over 6 days (numbered from 1 to 6). The side on which the olfactory stimulus was presented (left or right) was randomly distributed and balanced. The order in which the birds were tested was randomly chosen using sample function in R 3.1.2 (R Development Core Team 2015) software. After each 10-min trial, the observer (S.P.) opened the ball containing the meat, in front of the bird, and gave the meat to the bird. The observer (S.P.), who analyzed the videos, was blind to the side of the olfactory stimulus and the number (day) of the trial. Indeed, each video was renamed by a naïve person before the analysis. The time (in seconds) that each bird spent in contact with the control and smelling ball (i.e., with the beak or a foot in contact with the ball), the number of contacts with each ball, the first choice and the latency before the animal interacted with the stimulus were measured from the video recordings.

### Experiment 2: visual or olfactory cue

One month before the experiment, four rice cooking balls were painted with a matt varnish acrylic paint spray (AMT3760080621171, AMT, Orléans, France). After 2 days of drying, two of the balls were painted with a green “anise” acrylic paint spray (AMT3760080621225, AMT, Orléans, France) and other two with a blue “chekington” acrylic paint spray (AMT3760080621263, AMT, Orléans, France) (Fig. S1). These colors were chosen for two reasons. First, raptors (e.g., Eurasian sparrowhawks *Accipiter nisus* and common buzzards *Buteo buteo*) are generally considered to be tetrachromatic, with maximum spectral sensitivity in the violet, red, green and blue wavelengths (Jones et al. 2007; Lind et al. 2013). Second, we wanted to avoid using yellow (the color of the chicks fed to the birds) and red (the color of meat), as this may have resulted in an association between vision and olfaction due to previous experience, for example, humans systematically match certain odors to specific colors (Demattè et al. 2006; Gilbert et al. 1996).

#### Training

Each training session lasted 5 days. For each session, two rice cooking balls (one green and one blue) were presented to the birds. Each day we conducted one session of 1 min for each bird. During these sessions, each bird was presented with the two closed balls (that birds could not open) and was trained to eat [after the observer (S.P.) opened the ball at the end of the session] from a colored ball containing the meat. Different colored balls were attributed to different birds (Table S1). The side of the olfactory/visual stimulus was randomly distributed and balanced, as was the order in which birds were used, using sample function in R 3.1.2 (R Development Core Team 2015) software.

#### Testing

On the sixth day, each bird performed one 10-min session. For this session, the piece of meat was associated with the opposite colored ball to that used during the training phase (cue–conflict association). The side of the olfactory/visual stimulus was randomly distributed and balanced, as well as the order in which birds were used. The observer (S.P.), who was blind to the identity of the visual and olfactory stimuli, measured the time that each bird spent in contact with both balls and the number of contacts with each ball, as described above.

### Statistical analyses

Analyses were carried out using R 3.1.2 (R Development Core Team 2015) using {lmerTest} (Kuznetsova et al. 2017), {lsmeans} (Lenth 2016), {RVAideMemoire} (Hervé 2016), {coin} (Hothorn et al. 2008) and the {ggplot2} (Wickham 2016) packages. Throughout the paper, means are presented ± SE.

#### Experiment 1

We used a step approach to analyze the data from simple and easily understandable tests on counts to more sensitive tests. We first compared, for each species independently, the time spent in contact and the number of contacts with each ball with a permutation test for dependent paired samples. We next tested for both species together the effect of several explanatory variables on the time spent in contact and the number of contacts using generalized linear mixed models (GLMM). Among all the explanatory variables used [i.e., stimulus (balls), side, trials, species and the interactions between (a) stimulus and trials, (b) stimulus and species], we selected the best fixed-effect structure based on AICc criterion using the mod.sel function from the {MuMIn} package (Barton and Barton 2012) (see Table S2 supplementary materials for model selection based on AICc criterion). Individual identity nested within species was included as a random factor. To make post-hoc comparisons when significant interactions were found, we performed pairwise comparisons with Tukey adjustment of *p* values. We inspected the residuals of each model to ensure that they fitted the assumptions of GLMMs. We fitted a GLMM using Gaussian error distribution for the time spent in contact. For the number of contacts, we fitted a GLMM using Poisson error distribution.

The first choice of individual at every trial was analyzed using GLMM following a binomial error distribution. Trials, side and species identity were added as fixed effect. We used GLMM following a Gaussian error distribution to analyze the latency before the animal interacted with the stimulus with trials, side, success of first choice and species identity as fixed effect. In both models, individual nested in species was added as random effect.

We also measured the behavioral exploration in the aviaries during the experiments. The exploration behavior was defined as the number of switches between interactions. For experiment 1, we compared the difference in exploration behavior between species with GLMM following a “Poisson” error distribution. Individual nested in species was added as random effect.

#### Experiment 2

We compared time spent in contact, number of contacts with both balls and latency to make the first choice with a non-parametric Mann–Whitney–Wilcoxon test as no replication was done per individual. We used a Mann–Whitney–Wilcoxon test to compare differences in behavioral exploration in experiment 2.

#### Comparison experiment 1 and 2

To compare differences in behavioral exploration between the last trial of experiment 1 and experiment 2, we used a Mann–Whitney–Wilcoxon test. We took only the last trial of the Experiment 1 one to compare in similar conditions.

## Results

For both experiments, individuals interacted at least with one ball in every session.

### Experiment 1: olfactory ability

**Fig. 1:**
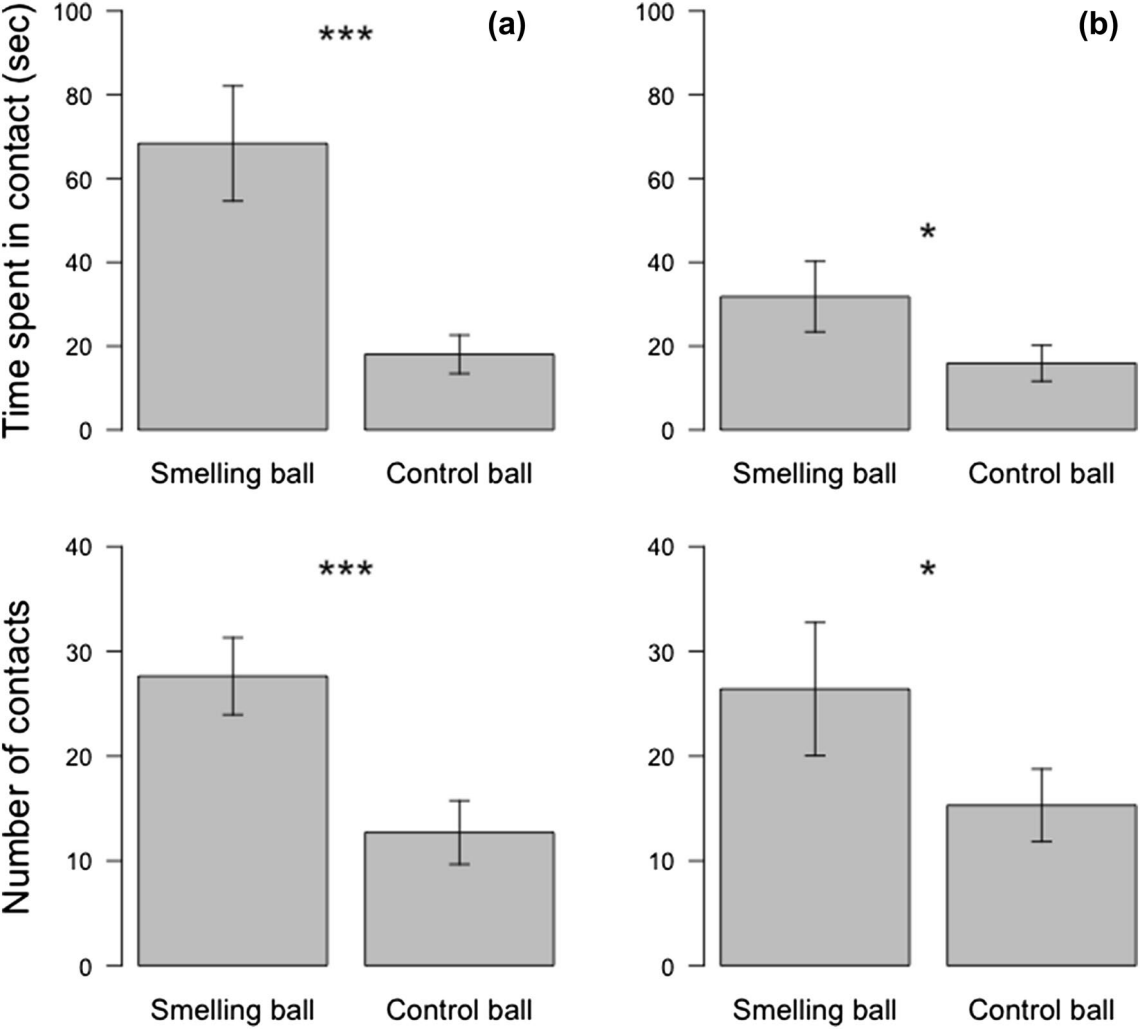
Time spent in contact and number of contacts with the smelling and control balls for **a** Turkey vulture and **b** Southern caracara in the experiment 1. Each bird performed a 10-min trial a day during 6 days. *p < 0.05 **p < 0.01 ***p < 0.001 (permutation test analyses)

For both species, we found that the time spent in contact differed significantly between balls. The time spent in contact with the smelling ball was four times longer (68.4 ± 13.7 vs. 18.0 ± 4.6 s) and two times longer (31.8 ± 8.4 vs. 15.0 ± 4.3 s) than with the control ball for the vultures (permutation test, *Z* = 3.11, *p* < 0.001) and the caracaras (permutation test, *Z* = 1.89, *p* = 0.029), respectively (Fig. 1; Movie 1). Individuals of both species made almost twice as many contacts with the smelling ball (vultures: 28 ± 4 vs. 13 ± 3, *Z* = 2.82, *p* = 0.002; caracaras: 26 ± 6 vs. 15 ± 3, *Z* = 1.67, *p* = 0.047), compared with the control (Fig. 1; Movie 1).

**Fig. 2:**
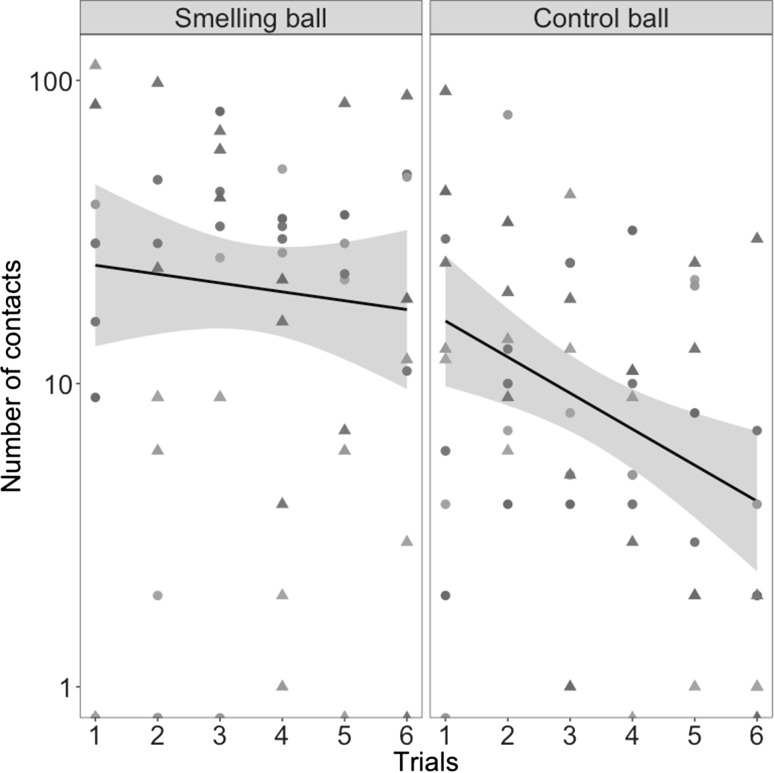
Number of contacts with smelling and control stimulus balls for both species together. The line and shaded area represent the regression and 95% confidence interval. Each symbol represents a different species (dots: Turkey vultures; triangles: Southern caracaras) and each color represents a different individual within a species (five individuals per species)

With analyses from the more sensitive GLMM analysis regrouping both species, we found a significant interaction between species and stimulus (Table 1). After post-hoc test comparisons, it appeared that the difference in the time spent in contact with both ball was significant for vultures (*t* = 4.257, *p* < 0.001) but not for caracaras (*t* = 1.419, *p* = 0.482). The difference in time spent in contact was not dependent upon the side of smelling ball as well as the trial rank (Table 1). The number of contacts with both stimuli differed significantly between species (Table 1) but both species made more contacts with the smelling balls than the control balls (Post-hoc comparisons, *p* < 0.001 for both species). For both species together, we recorded a decrease in the number of contacts with the control but not the smelling ball over trials (Table 1; Fig. 2).

**Table 1:** Results of selected generalized linear mixed models used to evaluate the effects of treatments (stimulus, trials, side and species) on behavioral variables

Trait analyzed	Variable	Estimate	Std. error	*t* (or *z*)*	*p* value
Time spent in contact	Intercept	68.810	10.340	6.655	< 0.001
	Side food	− 14.470	7.972	− 1.815	0.078
	Species × stimulus	34.420	16.300	2.111	0.035
Number of contacts	Intercept	3.408	0.244	13.986	< 0.001
	Side food	− 0.185	0.042	− 4.365	< 0.001
	Species × stimulus	0.251	0.088	2.855	0.004
	Trial × stimulus	− 0.240	0.027	− 8.748	< 0.001
First choice	Intercept	− 0.894	0.396	− 2.259	0.008
	Side food	2.643	0.671	3.940	< 0.001
	Species	1.218	0.683	1.784	0.074

*Depending on the distribution law implemented in the GLMM (*t* for Gaussian distribution and *z* for poisson and binomial distributions)

At 4 m distance (i.e., the starting perch), individuals walked straight to whichever ball was their first choice. Individuals (independently of the species identity) preferentially choose the smelling ball first [72.4% (42/58), GLMM, Table 1]. We also found that individuals preferentially choose the ball placed on the left side first [77.6% (45/58), GLMM, Table 1].

The latency in making the first choice (26.3 ± 4.5 s) was independent of the side where the smelling ball was placed, the success in the first choice, the trial rank and the species identity (all *p* > 0.1).

The vultures explored the balls more than the caracaras (21 ± 4 vs. 9 ± 1 number of switches between interactions for vultures and caracaras respectively; GLMM, *z* = − 2.981, *p* = 0.003).

### Experiment 2: visual or olfactory cue

In the test phase, most individuals first went to the colored ball that they had used during training sessions on the previous five consecutive days (5/8 vultures and 6/8 caracaras). The latency in making the first choice was significantly higher in the caracaras (27 ± 6 s) than the vultures (9 ± 3 s) (Wilcoxon test, *N* = 16, *W* = 55, *p* = 0.015).

**Fig. 3:**
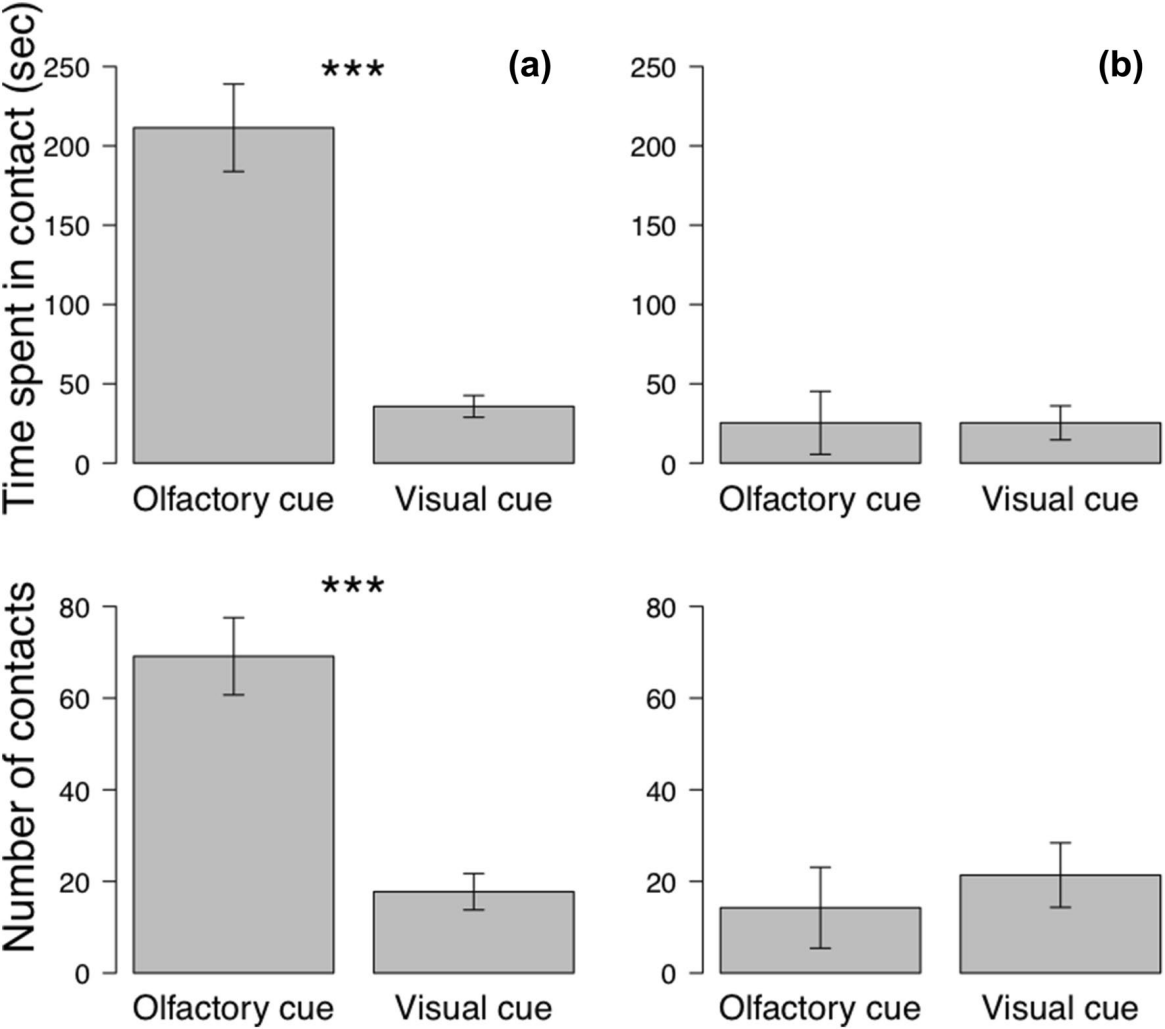
Time spent in contact and number of contacts with the olfactory and visual stimuli for **a** Turkey vulture and **b** Southern caracara in the experiment 2. *p < 0.05 **p < 0.01 ***p < 0.001

The time spent in contact with the smelling ball was six times longer than with the colored ball previously associated with food for the vultures (Wilcoxon test, *N* = 8, 211.38 ± 27.56 vs. 35.75 ± 6.86 s; *W* = 64, *p* < 0.001; Fig. 3), but no difference was found for the caracaras (Wilcoxon test, *N* = 8, 25.37 ± 19.87 vs. 25.38 ± 10.71 s; *W* = 21, *p* = 0.27, Fig. 3).

The vultures made four times more contacts with the smelling ball compared with the colored ball previously associated with food (Wilcoxon test, *N* = 8, 69 ± 8 vs. 18 ± 4 contacts; *W* = 63, *p* = 0.001, Fig. 3), but no differences were found for the caracaras (Wilcoxon test, *N* = 8, 14 ± 9 vs. 21 ± 7 contacts; *W* = 20, *p* = 0.22, Fig. 3).

The vultures explored the balls more than the caracaras (9 ± 1 vs. 3 ± 1 number of switches between interactions for vultures and caracaras respectively; Wilcoxon test, *W* = 62, *p* = 0.002).

### Comparison experiment 1 and 2

There was no significant difference in exploration behavior between the last trial of experiment 1 and experiment 2 for the vultures (Wilcoxon test, *W* = 25, *p* = 0.509) and for the caracaras (Wilcoxon test, *W* = 27.5, *p* = 0.292).

## Discussion

In this study, we found clear evidence that both Turkey vultures and Southern caracaras can smell, and thus find, the hidden food. However, while it appeared that the sense of smell may be a primary foraging sense in vultures, at least in our experimental setup, this was not the case for the Southern caracaras.

In experiment 1, we found that Turkey vultures and Southern caracaras can smell hidden odorous food both at 4 m distance and when they are close to the source of the odor, suggesting that both species may use olfaction to forage, at least at 4 m distance. In vultures (permutation and GLMM tests) and caracaras (permutation tests), time spent in contact and the number of contacts with the smelling ball were found to be significantly higher than with the control ball. In our experiment, while vultures used their beak to interact with the balls (and sometimes kept the balls for long time in their beak), caracaras interacted with the balls using their feet, as they naturally do (Del Hoyo and Elliot 1994), and made only brief contacts. Thus, an increase in the number of contacts with the smelling ball may have led to a relatively small increase for the exact time spent in contact not detected by the more sensitive GLMM. Moreover, the overall significant decrease in the number of contacts with the control ball (but not with the smelling ball) over trials for both species strongly suggests that individuals relied on olfaction to focus their attention to the ball containing hidden food. Therefore, in addition to the absence of neophobia (these captive individuals had never previously been fed with beef meat), this suggests that over multiple trials, individuals may have learned to recognize the unfamiliar food using their olfactory sense only. Indeed, because falconers only trained these captive birds with visual cues, their olfactory sense is not required during their training routines. In contrast, in our experiment, birds may have learned to rely on their olfactory sense as opposed to their visual sense.

In experiment 2, we used a similar protocol, but when both olfactory and visual cues were available, olfaction seems to be the predominant sense in Turkey vultures. This predominance is suggested by the fact that vultures rapidly changed their foraging behavior and relied on olfaction despite the fact that they were daily trained to use vision by the falconers. By contrast, Southern caracaras seem to rely on both olfactory and visual cues. Unfortunately, it was impossible to ensure that the birds were conditioned to the visual cue (color) during the training sessions because both visual and olfactory cues were associated, and thus inseparable (birds could see at the same time the color of the ball, and also smell the meat), for the purpose of the experiment. Nevertheless, the highest number of first choices toward the visual cue and the different pattern for caracaras between experiment 1 and 2 strongly suggested that they associated the colored ball with the meat.

In the wild and forested environments, Turkey vultures search for food mainly flying over the forest (Houston 1986), meaning that food sources are presumably hidden from sight by the canopy. However, field observations report that Turkey vultures are the first raptors to find carrion in tropical forests (Houston 1986, 1988). While we tested the olfactory ability at maximum 4 m distance, we showed that Turkey vultures may indeed use olfaction for foraging and finding entire carcasses that produce smell much more intense than the lure used in our experiment. In addition, our results show that olfaction seems to play a more significant role than color vision in foraging in Turkey vultures. In experiment 2, the color of the potential food container was disregarded once birds explored the balls closely. In spite of the fact that the ball containing smelly food had a different color than the color to which they were accustomed to find food in, vultures persistently inspected and tried to open the smelling ball, independent of its color. While color vision has not been demonstrated for either species, vultures may not rely as heavily on vision as other raptor species when foraging. Indeed, physiological and anatomical data show that Turkey vultures have relatively low visual acuity (Lisney et al. 2013) compared to other raptors such as eagles, falcons, hawks and even other vulture species (Fischer 1969; Hirsch 1982; Inzunza et al. 1991; Jones et al. 2007; Potier et al. 2016a, b; Reymond 1985, 1987). By contrast, Turkey vultures do have relatively large olfactory bulbs compared to other raptors (Cobb 1968; Grigg et al. 2017). We can therefore reasonably hypothesize that this species may not rely as heavily on vision as other raptor species when foraging, and rather may rely more on olfaction.

In comparison, Southern caracaras seem to have higher visual acuity. Indeed, the visual acuity of a closely related species, the Chimango caracara *Phalcoboenus chimango* (with a mean of 28.5 cycles per degree, i.e., they can see 28.5 cycles (consisting of one black and one white bar) in one degree of visual angle) (Potier et al. 2016a), is approximately twice as high as that of the Turkey vulture (15.6 cycles per degree) (Lisney et al. 2013). In the wild and forested environment, caracaras search for food mainly by walking around under the forest canopy (Sazima 2007). Caracaras forage on the ground opportunistically, and in some cases, they also feed on prey with a strong odor emission, such as carcasses or insects found in dung (Del Hoyo and Elliot 1994). Therefore, caracaras might switch between different senses according to the ecological circumstances, or the specific requirements of identifying particular food sources. This strategy would reduce the time and effort spent foraging. Our study supports this hypothesis. We found that caracaras interacted significantly more with the smelling ball and tried to open it, when only olfactory cues were available to them, suggesting that these opportunistic feeders may rely on smell to find hidden food. To the best of our knowledge, this is a first for a falconid bird. While a recent study showed that three other falconids, the Peregrine falcon *Falco peregrinus*, the Saker falcon *Falco cherrug*, and the Gyrfalcon *Falco rusticolus*, avoided or rejected food when the odor of a foul-smelling secretion of the great spotted cuckoo (*Clamator glandarius*) was sprayed on a food reward (Röder et al. 2014), it is not clear from this study whether the repellent effect was due to the taste or the scent, as the authors vaporized the secretion directly on the food.

In contrast to Turkey vultures, olfaction did not appear to be more important than sight (or vice versa) in Southern caracaras. In fact, in the second experiment, where the relative importance of olfactory and visual cues was tested, Southern caracaras did not show any preference, interacting similarly with the two colored balls even though only one of them was also odorous. To explain this result, we can suggest two non-exclusive hypotheses. First, Southern caracaras, as hypothesized by observations in the wild, may opportunistically use both olfaction and vision to find food: in our experiment, both the colored and smelling balls bore cues indicating the presence of food, leading them to check both stimuli alternatively. Indeed, in our experimental setup, they had learned previously to find food in a given colored ball, but were perceiving the food’s odor coming from the differently colored ball. This opportunistic use of different senses in different behavioral contexts has been shown in other bird species. For example, American robins (*Turdus migratorius*) search for earthworms using acoustic cues when visual cues are limited (Heppner 1965; Montgomerie and Weatherhead 1997). The second hypothesis may be that the experimental setup confused the birds between the training and the test phase, leading individuals to randomly explore the two balls as they were familiarized to feed from a specific colored ball, but smelled food in the other one. This leads to an apparent inconsistency between a stored information and a perception. However, this alternative explanation does not change the ecological value of our findings.

Our study suggests that species with different ecological niches can rely on different senses according to specific ecological contexts and/or circumstances, but also according to their distinct evolutionary pathways. Indeed, it is important to note that the two raptor species used in this study, Turkey vultures and Southern caracaras, come from two different avian orders (Accipitriformes and Falconiformes, respectively), which are drawn from distinct evolutionary pathways (Jetz et al. 2012; Prum et al. 2015). These distinct pathways may have led to different olfactory abilities, evidenced by the different olfactory bulb size among bird orders (Corfield et al. 2015).

Similar results have been reported for other animals. For example, Wells and Lehner (1978) found that in coyotes (*Canis latrans*) foraging for rabbits under experimental conditions, although vision, audition and olfaction can each be used alone, vision seemed to be the predominant sense. Another example is that of Cory’s shearwaters, which home to their nest burrow at night in almost all known colonies, presumably relying on olfaction only (Dell’Ariccia and Bonadonna 2013). In birds, information on the relative importance of olfaction and vision is scarce and has been only studied in procellariiformes (Mardon et al. 2010; Nevitt et al. 2008) and homing pigeons (Wallraff 2005). The main explanation for this is certainly that the relative importance of olfaction in most birds has historically been vastly underestimated (Roper 1999). Exploring these unsuspected sensory abilities may help us solve former unanswered questions in avian behavioral ecology, such as how Old World vultures can detect buried carrion (Gilbert and Chansocheat 2006). Another such example is the perception of sugar taste in hummingbirds: while behavioral experiments suggested that these birds can taste sugar (del Rio 1990), this observation was rejected because birds lack the conventional sweet taste receptors genes found in other vertebrates. This paradox was finally unraveled when Baldwin et al. (2014) discovered that hummingbirds actually do possess a sweet taste receptor, which remained unknown because it is transformed from the ancestral umami receptor. In conclusion, as scientists we sometimes have the tendency to focus on “what is known”, while the incredible diversity of sensory abilities of animals is not obvious from a human perspective.

## Electronic supplementary material

Below is the link to the electronic supplementary material.

Supplementary material 1 (MP4 9614 KB)

Supplementary material 2 (DOCX 4428 KB)
