# Supplementary material for: Sight or smell: which senses do scavenging raptors use to find food?
Source: Anim Cogn. 2018 Oct 26;22(1):49–59. doi: 10.1007/s10071-018-1220-0 (PMC6326982; doi:10.1007/s10071-018-1220-0)
Supplement: Supplementary file 2 — Supplementary material 2 (DOCX 4428 KB) [file 10071_2018_1220_MOESM2_ESM.docx]

**Supplementary materials**

**Sight or smell: which senses do scavenging raptors use to find food?**

Simon Potier^a,b*^, Olivier Duriez^a^, Aurélie Célérier^a^, Jean-Louis Liegeois^c^ and Francesco Bonadonna^a^

^a^ CEFE UMR 5175, CNRS - Université de Montpellier - Université Paul-Valéry Montpellier - EPHE – 1919 route de Mende, 34293 Montpellier cedex 5, France

^b^ Department of Biology, Lund University, Sölvegatan 35, Lund S-22362, Sweden

^c^ Académie de Fauconnerie du Grand Parc du Puy du Fou, CS 70 025, 85590 Les Epesses

* Corresponding author: [sim.potier@gmail.com](mailto:simon.potier@cefe.cnrs.fr) / +336 11 31 67 16 / ORCID : [0000-0003-3156-7846](https://orcid.org/0000-0003-3156-7846)

**Table S1** Information on birds used in the experiments

| Species | ID | Birth year | Sex | Site | Month and year of experiment 1 | Month and year of experiment 2 | Colour of the ball associated with the smelling food in the training phase (experiment 2) | Weight in aviary (g) | Weight in experiment (g) | Weight variation (%) |
| --- | --- | --- | --- | --- | --- | --- | --- | --- | --- | --- |
|  |  |  |  |  |  |  |  |  |  |  |
| Turkey vulture | TV1 | 2001 | Unknow | 1 | April 2014 | September 2015 | Blue | 1840 | 1450 | -26.90 |
|  | TV2 | 2003 | Unknow | 1 | April 2014 | September 2015 | Blue | 1620 | 1350 | -20.00 |
|  | TV3 | 2003 | Unknow | 1 | October 2014 | September 2015 | Green | 1680 | 1450 | -15.86 |
|  | TV4 | 2010 | Unknow | 1 | October 2014 | September 2015 | Green | 1740 | 1350 | -28.89 |
|  | TV5 | 2010 | Unknow | 1 | April 2014 | - | - | 1900 | 1450 | -31.03 |
|  | TV6 | 2012 | Female | 2 | - | March 2016 | Green | 1490 | 1640 | +9.15 |
|  | TV7 | 2012 | Female | 2 | - | March 2016 | Green | 1480 | 1610 | +8.07 |
|  | TV8 | 2010 | Male | 2 | - | March 2016 | Blue | 1660 | 1760 | +5.68 |
|  | TV9 | 2011 | Male | 2 | - | March 2016 | Blue | 1490 | 1640 | +9.15 |
|  |  |  |  |  |  |  |  |  |  |  |
| Southern caracara | SC1 | 1990 | Male | 1 | October 2014 | September 2015 | Green | 1000 | 800 | -25.00 |
|  | SC2 | 2005 | Female | 1 | October 2014 | September 2015 | Blue | 1240 | 1000 | -24.00 |
|  | SC3 | 2011 | Male | 1 | October 2014 | September 2015 | Green | 940 | 850 | -10.59 |
|  | SC4 | 2012 | Female | 1 | October 2014 | September 2015 | Blue | 1100 | 950 | -15.79 |
|  | SC5 | 2012 | Male | 1 | October 2014 | - | - | 940 | 850 | -10.59 |
|  | SC6 | 2013 | Male | 2 | - | March 2016 | Blue | 850 | 930 | +8.60 |
|  | SC7 | 2009 | Male | 2 | - | March 2016 | Blue | 1000 | 970 | -3.09 |
|  | SC8 | 2007 | Male | 2 | - | March 2016 | Green | 1090 | 1080 | -0.93 |
|  | SC9 | 2007 | Male | 2 | - | March 2016 | Green | 1070 | 1030 | -3.88 |


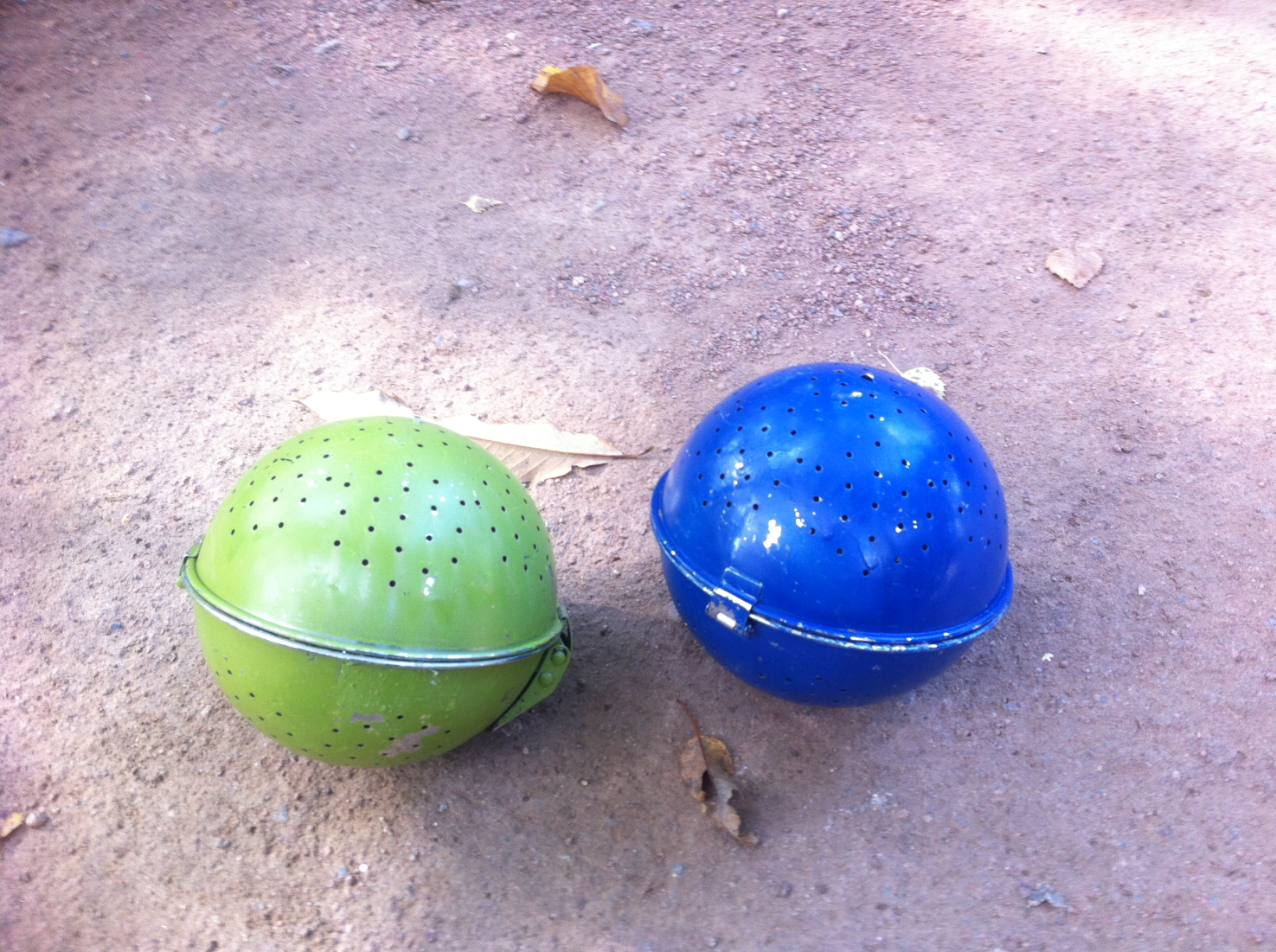


**Figure S1** Colored balls used in Experiment 2

**Figure S2** Experimental setup used in experiment 1 and 2. From a starting perch (A), each bird (here a southern caracara) has to choose between two rice cooking balls (B, C) presented at 4m from the starting perch and distant from 6m. Both rice cooking balls were placed in a plastic recipient.

**A**


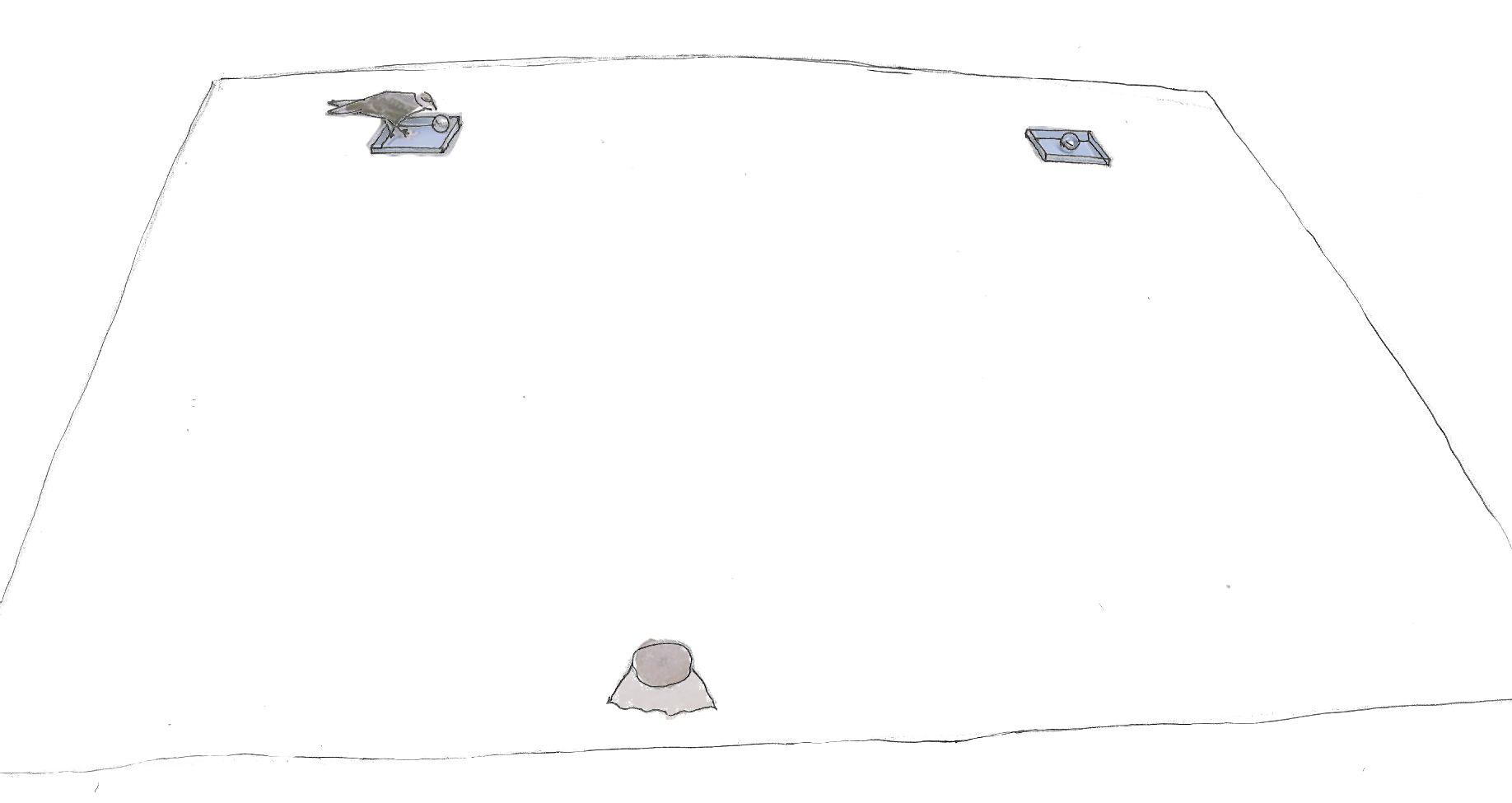


**B**

**C**

**Table S2** Model selection based on AICc criterion. Models presentation were expressed by specifying a confidence set of models (delta AICc less than 5)

| **Time spent in contact** |  |  |  |  |  |
| --- | --- | --- | --- | --- | --- |
|  |  |  |  |  |  |
| **Model** | **K** | **logLik** | **AICc** | **delta** | **weight** |
| Stimulus * species + side + (1 \| species:individual) | 7 | -591.71 | 1198.47 | 0.000 | 0.306 |
| Stimulus * species + (1 \| species:individual) | 6 | -593.33 | 1199.44 | 0.976 | 0.188 |
| trial + side + Stimulus * species + (1 \| species:individual) | 8 | -591.69 | 1200.76 | 2.293 | 0.097 |
| Stimulus + side + species + (1 \| species:individual) | 6 | -594.00 | 1200.78 | 2.309 | 0.096 |
| Stimulus + side + (1 \| species:individual) | 5 | -595.41 | 1201.37 | 2.904 | 0.072 |
| Stimulus + species + (1 \| species:individual) | 5 | -595.56 | 1201.67 | 3.198 | 0.062 |
| Stimulus * species + trial + (1 \| species:individual) | 7 | -593.32 | 1201.70 | 3.230 | 0.061 |
| Stimulus * trial + side + Stimulus * species + (1 \| species:individual) | 9 | -591.28 | 1202.29 | 3.820 | 0.045 |
| Stimulus + (1 \| species:individual) | 4 | -597.05 | 1202.47 | 3.999 | 0.041 |
| Stimulus + side + species + trial + (1 \| species:individual) | 7 | -593.99 | 1203.03 | 4.561 | 0.031 |
|  |  |  |  |  |  |
| **Number of contacts** |  |  |  |  |  |
|  |  |  |  |  |  |
| **Model** | **K** | **logLik** | **AICc** | **delta** | **weight** |
| Stimulus * trial + side + Stimulus * species + (1 \| species:individual) | 8 | -1163.29 | 2343.95 | 0.00 | 0.87 |
| Stimulus * trial + side + (1 \| species:individual) | 6 | -1167.49 | 2347.76 | 3.81 | 0.13 |
|  |  |  |  |  |  |
| **First choice** |  |  |  |  |  |
|  |  |  |  |  |  |
| **Model** | **K** | **logLik** | **AICc** | **delta** | **weight** |
| side + species + (1 \| species:individual) | 4 | -28.29 | 65.34 | 0.000 | 0.481 |
| side + (1 \| species:individual) | 3 | -30.00 | 66.45 | 1.109 | 0.276 |
| trial + side + species + (1 \| species:individual) | 5 | -28.23 | 67.62 | 2.280 | 0.154 |
| trial + side + (1 \| species:individual) | 4 | -29.99 | 68.73 | 3.396 | 0.088 |
|  |  |  |  |  |  |
| **Latency** |  |  |  |  |  |
|  |  |  |  |  |  |
| **Model** | **K** | **logLik** | **AICc** | **delta** | **weight** |
| 1 + (1 \| species:individual) | 3 | -276.27 | 559.01 | 0.000 | 0.235 |
| trial + (1 \| species:individual) | 4 | -275.82 | 560.42 | 1.412 | 0.116 |
| species + (1 \| species:individual) | 4 | -275.84 | 560.47 | 1.458 | 0.114 |
| side + (1 \| species:individual) | 4 | -275.97 | 560.73 | 1.720 | 0.100 |
| Stimulus + (1 \| species:individual) | 4 | -276.01 | 560.81 | 1.805 | 0.096 |
| trial + species + (1 \| species:individual) | 5 | -274.93 | 561.05 | 2.043 | 0.085 |
| Stimulus + species + (1 \| species:individual) | 5 | -275.40 | 562.00 | 2.990 | 0.053 |
| side + species + (1 \| species:individual) | 5 | -275.47 | 562.15 | 3.139 | 0.049 |
| Stimulus + trial + (1 \| species:individual) | 5 | -275.62 | 562.43 | 3.422 | 0.043 |
| trial + side + (1 \| species:individual) | 5 | -275.62 | 562.44 | 3.431 | 0.042 |
| Stimulus + species + trial + (1 \| species:individual) | 6 | -274.51 | 562.73 | 3.716 | 0.037 |
| Stimulus + side + (1 \| species:individual) | 5 | -275.92 | 563.04 | 4.030 | 0.031 |
